# Conservation of the separase regulatory domain

**DOI:** 10.1186/s13062-018-0210-0

**Published:** 2018-04-27

**Authors:** Michael Melesse, Joshua N. Bembenek, Igor B. Zhulin

**Affiliations:** 1 Department of Biochemistry, Cellular and Molecular Biology, University of Tennessee, Knoxville, TN 37996 USA; 2 Department of Microbiology, University of Tennessee, 1414 Cumberland Ave, Knoxville, TN 37996 USA; 3 Computational Sciences and Engineering Division, Oak Ridge National Laboratory, Oak Ridge, TN 37831 USA

**Keywords:** Separase, Conservation, PSI-BLAST, Cysteine motif

## Abstract

**ᅟ:**

We report a protein sequence analysis of the cell cycle regulatory protease, separase. The sequence and structural conservation of the C-terminal protease domain has long been recognized, whereas the N-terminal regulatory domain of separase was reported to lack detectable sequence similarity. Here we reveal significant sequence conservation of the separase regulatory domain and report a discovery of a cysteine motif (CxCxxC) conserved in major lineages of Metazoa including nematodes and vertebrates. This motif is found in a solvent exposed linker region connecting two TPR-like helical motifs. Mutation of this motif in *Caenorhabditis elegans* separase leads to a temperature sensitive hypomorphic protein. Conservation of this motif in organisms ranging from *C. elegans* to humans suggests its functional importance.

**Reviewers:**

This article was reviewed by Lakshminarayan Iyer and Michael Galperin.

**Electronic supplementary material:**

The online version of this article (10.1186/s13062-018-0210-0) contains supplementary material, which is available to authorized users.

## Findings

**Fig. 1:**
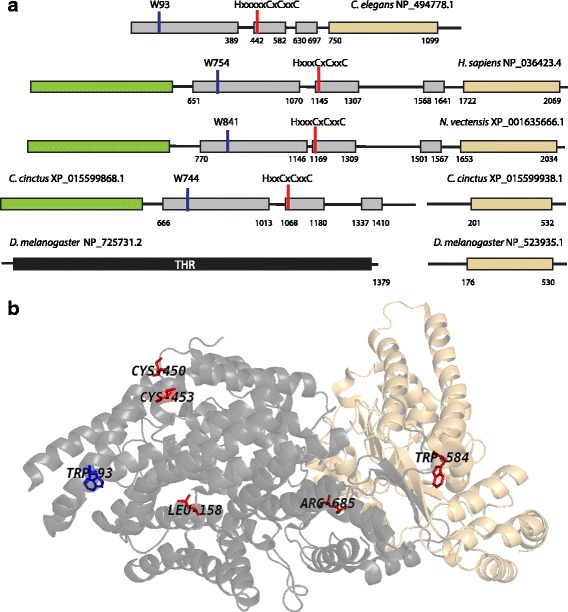
Domain architecture of separases from representative metazoan genomes and universally conserved positions in the separase regulatory domain. (**a**) The α-solenoid regulatory domain is depicted in gray and C-terminal protease domain is shown in light orange. The N-terminal α-helical domain missing from nematode sequences, but conserved in separases from most metazoans, plants, and fungi (except for *Saccharomycetes*), is shown in green. *D. melanogaster* THR protein is shown in black. (**b**) Universally conserved N-terminal residues are shown on the *C. elegans* separase Cryo-EM structure (PDB 5MZ6). The two visible conserved residues that make up the cysteine motif (C450 and C453) are shown in red and are found as part of a solvent exposed loop between helices 15 and 16, while neither C448 nor H442 were resolved in the crystal structure. Another universally conserved residue, W93, is shown in blue

Separase is a CD clan cysteine protease that regulates cell division. Separase proteolytic activity is regulated mainly by the binding of an inhibitory chaperone, securin [1, 2], which is degraded at anaphase onset in a proteasome dependent manner after polyubiquitination by the Anaphase Promoting Complex/Cyclosome [3, 4]. Once activated, separase cleaves a subunit of cohesin, allowing sister chromatids to segregate to opposite poles [5–8]. Subsequently, separase cleaves a number of other substrates to regulate several anaphase events [9–13]. Separase is functionally conserved in diverse organisms, including vertebrates, nematodes, fungi, and plants [14]. Conservation was reported only in the sequence [15–17] and structure of the C-terminal protease domain [18–20]. A large N-terminal regulatory domain of separase (Fig. 1) has been poorly characterized. No sequence conservation within this domain was detected [15] and N-termini of yeast and *C. elegans* separases are structurally different [19, 20]. In the *C. elegans* separase, the N-terminal α-solenoid domain consists of 25 α-helices, arranged as atypically compact TPR-like repeats [20, 21]. Various post-translational modifications of this domain regulate separase activity [22–26]; thus deeper insight into structure and function of this regulatory is needed.

No multiple sequence alignments of the separase N-terminal domains are available in the current literature; however, a pairwise sequence alignment between the human and nematode homologs was produced based on the recently solved three-dimensional structure of the *C. elegans* separase [20]. Twenty-five structurally identified helices in the N-terminal region of the *C. elegans* separase (NP_491160.1, aa 1–700) were aligned to the 25 α-helices predicted in the N-terminal region of the human separase (NP_036423.4, aa 651–1641). The very low percentage of identity between the two sequences and the fact that they were aligned manually, guided by structural information, prompted us to further explore potential sequence conservation in the separase regulatory domain.

In this study, we reveal significant sequence conservation of the separase regulatory domain and report a discovery of a cysteine motif (CxCxxC) conserved in major lineages of Metazoa including nematodes and vertebrates.

## Regulatory domain of separase is conserved

First, we performed a BLAST [27] search (with default parameters) of the non-redundant protein sequence database at the NCBI (NR) with the N-terminal (aa 1–740) portion of the *C. elegans* separase (accession NP_491160.1) as a query. The search confidently retrieved similar proteins from distantly related nematode species and reciprocal BLAST searches validated their orthology. Next, we performed exhaustive PSI-BLAST [28] searches (with default parameters) of the NR database with N-terminal regions of the nematode sequences as queries. The search initiated with the N-terminal region (aa 1–760) of the *Toxicara canis* separase (KHN86283.1) retrieved a separase from a vertebrate *Danio rerio* (XP_001337869.1) with E value 9e-04 in the third iteration and a human separase (NP_036423.4) with E value 2e-11 in the fourth iteration, among many other separase sequences from vertebrates. We then constructed a multiple sequence alignment of representative nematode and vertebrate sequences using MAFFT [29] with default parameters (Additional file 1). Overall, the alignment, which was only minimally edited based on structural information, looks similar to that of the *C. elegans* and *H. sapiens* sequences published by [20]; however, there are drastic differences with respect to conserved positions. While more than 80 identical residues in the *C. elegans* and *H. sapiens* sequences were identified by Boland et al. (Supplementary Fig. 4 in ref. [20]), our alignment shows only 7 such positions. The majority of identical positions revealed by comparing only two sequences do not match conservation patterns revealed by our multiple sequence alignment. These alignments appear to be due to incorrect gap placements or happen purely by chance (Additional file 1), which further emphasizes problems associated with building and interpreting “manual alignments”, even when assisted by structural information [30].

## Identification of the CxCxxC motif

Among the very few identical positions that we identified in nematode and vertebrate N-terminal sequences (Fig. 1), four are in various parts of the region: W93/W754 (*C. elegans/H. sapiens*), L158/L827, W584/W1294, and R685/R1629. The remaining three identical residues, C448/C1146, C450/C1148, and C453/C1151, appear to form a motif, which is located at the border of Insert 1 and helix H16 (Additional file 1). Closer examination revealed a histidine residue located 4–6 residues upstream of the first cysteine in this motif. Conservation of this motif in nematodes and vertebrates prompted a question about its broader phyletic distribution. Multiple BLAST and PSI-BLAST searches initiated with separase regulatory domains from vertebrates and nematodes revealed similar sequences in representatives of other metazoan phyla. These results are summarized in Additional file 2 and representative sequences are shown in Additional file 1. In brief, homologous domains were found in representatives of the following major metazoan clades: Cnidaria, Arthropoda, Priapulida, Mollusca, Brachiopoda, and Echinodermata. Satisfactorily, the CxCxxC motif was found in all of them at the expected location and in > 90% of cases, a histidine residue was located 4–6 residues upstream of the first cysteine in the motif. Furthermore, at least one other residue, a tryptophan located in the TPR1B repeat (Fig. 1, Additional file 1) appears to be universally conserved. While the function of this newly identified motif is unknown and both the histidine and first cysteine are not resolved in the crystal structure, the motif could be a metal binding site, e, g, as seen in various cysteine and histidine containing Zn-binding motifs [31].

We found no evidence of homology between the separase regulatory domains of vertebrates and non-metazoans as well as representatives of one metazoan phylum - Platyhelminthes (Additional file 2) and no CxCxxC motif was found in these sequences. It is important to note that the detailed analysis of separase phyletic distribution is hampered by a poor quality of gene calling in at least some cases. We failed to identify any separase genes in several genomes, where it was expected judging by its presence in closely related species (Additional file 2). Similarly, in some cases the regions where the CxCxxC motif was expected were missing from separase sequences, also likely due to incorrect gene calling (Additional file 2). Nonetheless, results obtained strongly suggest that the α-solenoid regulatory domain, which is present in the vast majority of metazoan separases, originated early in metazoan evolution and that the CxCxxC motif is the most conserved feature of this domain. A notable exception is the absence of this motif in a few lineages of Arthropoda. Functional separase in *D. melanogaster* is a product of two separate genes that encode proteins corresponding to the N-terminal region (THR) and the C-terminal domain (Sse) [32]. We found that this is true for some other, but not all Arthropoda (Additional files 2 and 3). In Crustacea, Merostomata, and Scorpiones, the protein corresponding to the N-terminal separase region is well conserved and contains the CxCxxC motif. In some Arachnida, separases are encoded by a single gene and contain the cysteine motif, but in others the N-terminus shows no similarity to classical separases and contains no cysteine motif (Additional file 2). Most interestingly, while the N-terminal separase protein and its CxCxxC motif are conserved in all major families of insects, this motif is missing from all flies of the suborder Brachycera, which includes *Drosophila* (Additional file 3). The corresponding protein, THR, has insignificant similarity (if any) to the classical separase. It was proposed to be homologous to the regulatory domain of a classical separase [32]; however, we could not find any evidence for homology. For example, searches originated with the THR sequences retrieve various proteins unrelated to separase from distantly related species, but fail to retrieve proteins corresponding to the separase N-terminal region from closely related Diptera. Thus, most likely, THR represents a case of a non-orthologous gene displacement. This protein performs a function analogous to that of the canonical regulatory domain [32], but the details of its interaction with the C-terminal separase domain could be quite different.

## *C. elegans* as a model to study the role of the CxCxxC motif

Our analysis shows that, in contrast to *D. melanogaster*, *C. elegans* is an excellent model to study the role of the separase regulatory domain, because it is orthologous to that in humans. As expected, multiple residues, distributed throughout this domain (Additional file 4), are conserved among nematode separases, which are different from residues conserved among vertebrate separases. None of these conserved residues are in contact with the separase inhibitory chaperone, securin. The nature of this conservation remains unclear. None of the highly conserved residues appear to form a contact with another similarly conserved residue, as judged by the proximity of beta carbons on the separase structure (Additional file 5). However, no changes in these residues are observed in human allelic variants or sequenced cancer populations implying that mutations in these resides may be lethal (Additional file 6). More importantly, a mutation in the newly identified CxCxxC motif (C450Y) of the *C. elegans* separase (*sep-1(e2406)*) results in a temperature sensitive phenotype that leads to exocytosis defects [12, 33]. We identified multiple intragenic suppressors of *sep-1(e2406)* that exclusively introduce mutations to the regulatory domain [34]. These positions are not conserved, do not contact securin and are distributed throughout the regulatory domain (Additional file 4). Understanding effects of these mutations will require further investigation, but our finding that the regulatory domain of separase has distinct conserved elements strongly supports its functional importance. In the future, it will be of great interest to determine the active conformation of separase and to investigate the functional role of this motif. Our analysis also demonstrates the utility of studies in *C. elegans* in understanding separase regulation in humans.

## Reviewers’ comments

### Reviewer 1: Lakshminarayan Iyer, National Institutes of Health

Melesse and colleagues report the presence of a conserved cysteine cluster in the N-terminal region of the separases. Per se the work is reproducible and the details of the motif and the N-terminal domain are well described. This study is of importance to researchers in the cell cycle/separase field. It is also very curious that a motif is retained between nematodes and vertebrates and not in other metazoan clades, as genome comparisons show that nematodes are usually fast evolving and often lose proteins and domains/motifs observed in vertebrates and other metazoaon clades. I do have a couple of minor comments and suggestions. 1. One of the earliest computational studies on the caspases noticed the TPR-like repeats at the N-terminus, and this might be worth citing (PMID: 11835511).

Author’s response: *Indeed, this is a notable finding, which predates the knowledge obtained by solving the 3D structure. This paper is cited in the revised manuscript.*

2. There is a histidine about 4–6 residues upstream of the triple cysteine motif in the nematode separases. Could they possibly align with the conserved Histidine 4 residues upstream of the vertebrate triple Cysteine motif? These might suggest a neomorphic metal-binding motif. This might also be confirmable by available structures.

Author’s response: *This is an interesting observation. Indeed, even in some unedited MAFFT alignments the histidines were matched at the expense of introducing a gap. Furthermore, in newly identified sequences from other metazoan phyla, a histidine is present in the same location (4–6 residues upstream of the triple cysteine motif). We now acknowledge this fact in the text and following the Reviewer’s suggestion propose that one potential role for this motif could be metal binding,* e.g.*, as seen in various cysteine and histidine containing Zn-binding motifs (Pace & Weerapana 2014). Unfortunately, both the histidine and the first cysteine residue of this motif are not resolved in the crystal structure.*

3. Was the cysteine cluster motif used to search a limited database of separases (either using a motif searching tool, or by HMMer or some such profile based method) to check if other metazoans might possess it in a comparable location?

Author’s response: *Following this question and suggestions from Reviewer 2, we identified N-terminal separase domains in representatives of other metazoan phyla and identified the three cysteine motif in those using a simple CxCxxC string search. Satisfactorily, (i) there was only one such motif in each of these sequences, (ii) in most (but not all) cases, a histidine residue was located 4–6 residues upstream of the motif, and (iii) these motifs were perfectly aligned by BLAST in pairwise comparisons and by MAFFT in multiple sequence alignments of full-length domains prior to any editing. Based on these results, we expanded our description of this motif and its occurrence in Metazoa.*

### Reviewer 2: Michael Galperin, National Institutes of Health

This paper describes an interesting attempt to investigate sequence conservation within vertebrate separases by comparing them to the recently studied protein from *C. elegans*. This work would benefit from addressing the following points. 1. The paper claims that the described CxCxxC motif of separase is conserved in nematodes and vertebrates. That is true but separase appears to have a much wider phylogenetic distribution. Separin-like proteins have been annotated in Lingula anatine (Brachiopoda), Hydra vulgaris and Exaiptasia pallida (Cnidaria), *Crassostrea gigas* (Mollusca), Apostichopus japonicas (Echinodermata), Hymenolepis microstoma (Platyhelminthes) and other organisms. Further, a simple BLAST search retrieves separase-like sequences in *Anoplophora glabripennis* (Insecta), Centruroides sculpturatus (Arachnida), *Acanthaster planci* (Echinodermata), and other invertebrates. The question then becomes, when did separase first evolve?

Author’s response: *Beside vertebrates and nematodes, separases have been previously described and experimentally studied in such diverse phyla as fungi (Ciosk R, et al.* [5], *Funabiki H, et al.* [35]*) and plants (Moschou PN, et al.* [36]*), although to our knowledge there was no study specifically addressing their evolutionary history. This was not a goal of our investigation either, but we agree with the Reviewer that it is important to place our specific motif discovery into a broader context of the separase phyletic distribution and our current understanding of its evolution. We have added more background information and our own observations related to this question. In brief, we identified separases in many other invertebrates, obtained evidence for their orthologous relationships and showed that in the majority of cases the N-terminal separase domain is recognizable and it contains the conserved CxCxxC motif at the same location.*

Is the described CxCxxC motif conserved in all invertebrate separases? If not, why?

Author’s response: *As our additional analysis shows, the CxCxxC motif is found in the vast majority of separase regulatory domains from most of the metazoan phyla. It is missing from all non-metazoan separases as well as from* Hemichordata*,* Tunicata, Placozoa*,* Porifera*, and* Platyhelminthes*, although only the latter phyla is represented by more than one genome, From this phyletic distribution, we can safely conclude that the separase N-terminal domain exemplified by vertebrate and nematode sequences originated fairly early in the metazoan evolution. It is likely impossible to answer the question why is this motif missing from some of the homologous N-terminal separase domains, especially because we do not know its function. Our best guess is that its function (whatever it is) can be either achieved or substituted by other means. Non-orthologous gene displacement of the entire separase N-terminal domain in a fly lineage is in line with this proposition.*

2. What is the importance of the described CxCxxC motif? Is it located at the separin interacting interface? Is there any evidence of metal binding or disulfide formation by any separases that have this motif?

Author’s response: *This is obviously the first report on the identification of this motif, so its function is yet to be determined. Its importance, however, is illustrated by the fact that mutation in this motif (C450Y) is highly damaging in C. elegans (Siomos* et al. *2001, Bembenek JN,* et al. *2007). The cysteine motif is located away from the known securin interacting and C-terminus interacting interfaces. As suggested by both reviewers, potentially this could be a metal binding motif; however, there is no evidence for this (or for disulfide bond formation) in the literature and no insight from the crystal structure, because half of this motif is not resolved. We hope that our finding will motivate the search for this motif function.*

3. The Additional file 4 should be moved into the main text. It should show the positions of the conserved Trp residues and the CxCxxC motif.

Author’s response: *We agree that showing the separase domain architecture with mapped conserved residues would be helpful to the reader. In the light of our new findings of a broader distribution of the cysteine motif, we now show a comparison of domain architectures for separases from several lineages as* Fig. 1a *(only one figure is allowed in the main text for a discovery note).*

## Additional files

Additional file 1: Figure: Multiple sequence alignment of separases from representative genomes. Sequences from 11 nematode species (top portion of each panel), from seven representative vertebrate species (human, opossum, turtle, chicken, frog, coelacanth, and fish) (middle portion of each panel), and from nine invertebrate species representing several other metazoan phyla (bottom portion of each panel) are shown. Twenty-five alpha helices (labeled H1 to H25) comprising 11 TPR-like repeats (labeled TPR1A,B to TPR11A,B) in the *C. elegans* separase (PDB accession 5MZ6) are shown above the alignment. Identical residues in each group are highlighted: negatively charged, red; positively charged, blue; aromatic, green; aliphatic, yellow; alcohol, magenta; small, grey. Universally conserved residues are highlighted with black boxes. NCBI accession numbers: Caenorhabditis_elegans_1, NP_491160.1; Caenorhabditis_brenneri_1, EGT38506; Caenorhabditis_briggsae_1, CAP33358; Caenorhabditis_remanei_1, XP_003114963.1; Loa_loa_1, XP_003140515.1; Wuchereria_bancrofti_1, EJW80934; Brugia_malayi_1, XP_001894870.1; Dictyocaulus_viviparus_1, KJH53363.1; Dictyocaulus_viviparus_2, KJH53362.1; Haemonchus_contortus_1, CDJ83415.1; Ancylostoma_duodenale_1, KIH65515.1; Ancylostoma_ceylanicum_1, EYC45610.1; Toxocara_canis_1, KHN86283.1; Homo_sapiens_1, NP_036423.4; Monodelphis_domestica_1, XP_007506592.1; Chelonia_mydas_1, XP_007058605.1; Gallus_gallus_1, XP_015128534.1; Xenopus_tropicalis_1, XP_004912005.1; Latimeria_chalumnae_1, XP_014347491.1; Maylandia_zebra_1, XP_014264400.1;; Acanthaster_planci_1, XP_022084422.1; Branchiostoma_floridae_1, XP_002607627.1; Priapulus_caudatus_1, XP_014674242.1; Lingula_anatina_1, XP_013410481.1; Crassostrea_gigas_1; XP_011423994.1; Lottia_gigantea_1, XP_009046347.1; Limulus_polyphemus_1, XP_022249257.1; Nematostella_vectensis_1, XP_001635666.1; Cephus_cinctus_1, XP_015599868.1. “Identical residues”* show positions defined as identical in a pairwise comparison of *C. elegans* and *H. sapiens* sequences by Boland et al., 2017. (PDF 64 kb)

Additional file 2: Table: Separases in representatives of major metazoan phyla. Products of two genes corresponding to N-terminal and C-terminal separase domains are highlighted in yellow and green, respectively. Truncated sequences are highlighted in grey. (XLSX 14 kb)

Additional file 3: Table: Proteins corresponding to the separase N-terminal domain in Insecta. Truncated sequence is highlighted in grey; sequences missing the CxCxxC motif are highlighted in yellow. (XLSX 15 kb)

Additional file 4: Figure: Separase N-terminal residues conserved among nematodes are distributed throughout the structure. *C. elegans* separase Cryo-EM structure (PDB 5MZ6) illustrating N-terminal residues conserved among nematodes found in the interior (**A**) and on the surface (**B**) of the TPR-like N-terminal domain. Intragenic suppressors of SEP-1(e2406) are shown (**C**) and are not among the conserved residues. The structures are oriented with the N-terminus to the left with a perspective that best illustrates the distribution of each highlighted residue. (TIF 926 kb)

Additional file 5: Table: Conserved residues found within the N-terminal domain helices. Residues that are within interacting distance (as assayed by measuring a distance less than 6 Å (Å) between β-Carbons) are indicated. These residues are generally located within the same helix and don’t appear to be important for stabilizing inter-helix interactions. (PDF 29 kb)

Additional file 6: Figure: Known mutations in human separase (ESPL-1). The collection of separase allelic variants of human Separase from the ExAC exome collection (http://exac.broadinstitute.org) and the ICGC (https://icgc.org/) which collects genomic sequences of various cancers. The frequency of each missense mutation is indicated. (TIF 413 kb)

## Abbreviations

BLAST: Basic local alignment search tool
MAFFT: Multiple alignment using fast fourier transform
PDB: Protein data bank
PSI-BLAST: Position-specific iterative BLAST
TPR: Tetratricopeptide repeat
