# Supplementary material for: Conservation of the separase regulatory domain
Source: Biol Direct. 2018 Apr 27;13:7. doi: 10.1186/s13062-018-0210-0 (PMC5921967; doi:10.1186/s13062-018-0210-0)
Supplement: Supplementary file 1 — Figure: Multiple sequence alignment of separases from representative genomes. Sequences from 11 nematode species (top portion of each panel), from seven representative vertebrate species (human, opossum, turtle, chicken, frog, coelacanth, and fish) (middle portion of each panel), and from nine invertebrate species representing several other metazoan phyla (bottom portion of each panel) are shown. Twenty-five alpha helices (labeled H1 to H25) comprising 11 TPR-like repeats (labeled TPR1A,B to TPR11A,B) in the C. elegans separase (PDB accession 5MZ6) are shown above the alignment. Identical residues in each group are highlighted: negatively charged, red; positively charged, blue; aromatic, green; aliphatic, yellow; alcohol, magenta; small, grey. Universally conserved residues are highlighted with black boxes. NCBI accession numbers: Caenorhabditis_elegans_1, NP_491160.1; Caenorhabditis_brenneri_1, EGT38506; Caenorhabditis_briggsae_1, CAP33358; Caenorhabditis_remanei_1, XP_003114963.1; Loa_loa_1, XP_003140515.1; Wuchereria_bancrofti_1, EJW80934; Brugia_malayi_1, XP_001894870.1; Dictyocaulus_viviparus_1, KJH53363.1; Dictyocaulus_viviparus_2, KJH53362.1; Haemonchus_contortus_1, CDJ83415.1; Ancylostoma_duodenale_1, KIH65515.1; Ancylostoma_ceylanicum_1, EYC45610.1; Toxocara_canis_1, KHN86283.1; Homo_sapiens_1, NP_036423.4; Monodelphis_domestica_1, XP_007506592.1; Chelonia_mydas_1, XP_007058605.1; Gallus_gallus_1, XP_015128534.1; Xenopus_tropicalis_1, XP_004912005.1; Latimeria_chalumnae_1, XP_014347491.1; Maylandia_zebra_1, XP_014264400.1;; Acanthaster_planci_1, XP_022084422.1; Branchiostoma_floridae_1, XP_002607627.1; Priapulus_caudatus_1, XP_014674242.1; Lingula_anatina_1, XP_013410481.1; Crassostrea_gigas_1; XP_011423994.1; Lottia_gigantea_1, XP_009046347.1; Limulus_polyphemus_1, XP_022249257.1; Nematostella_vectensis_1, XP_001635666.1; Cephus_cinctus_1, XP_015599868.1. “Identical residues”* show positions defined as identical in a pairwise comparison of C. elegans and H [file 13062_2018_210_MOESM1_ESM.pdf]

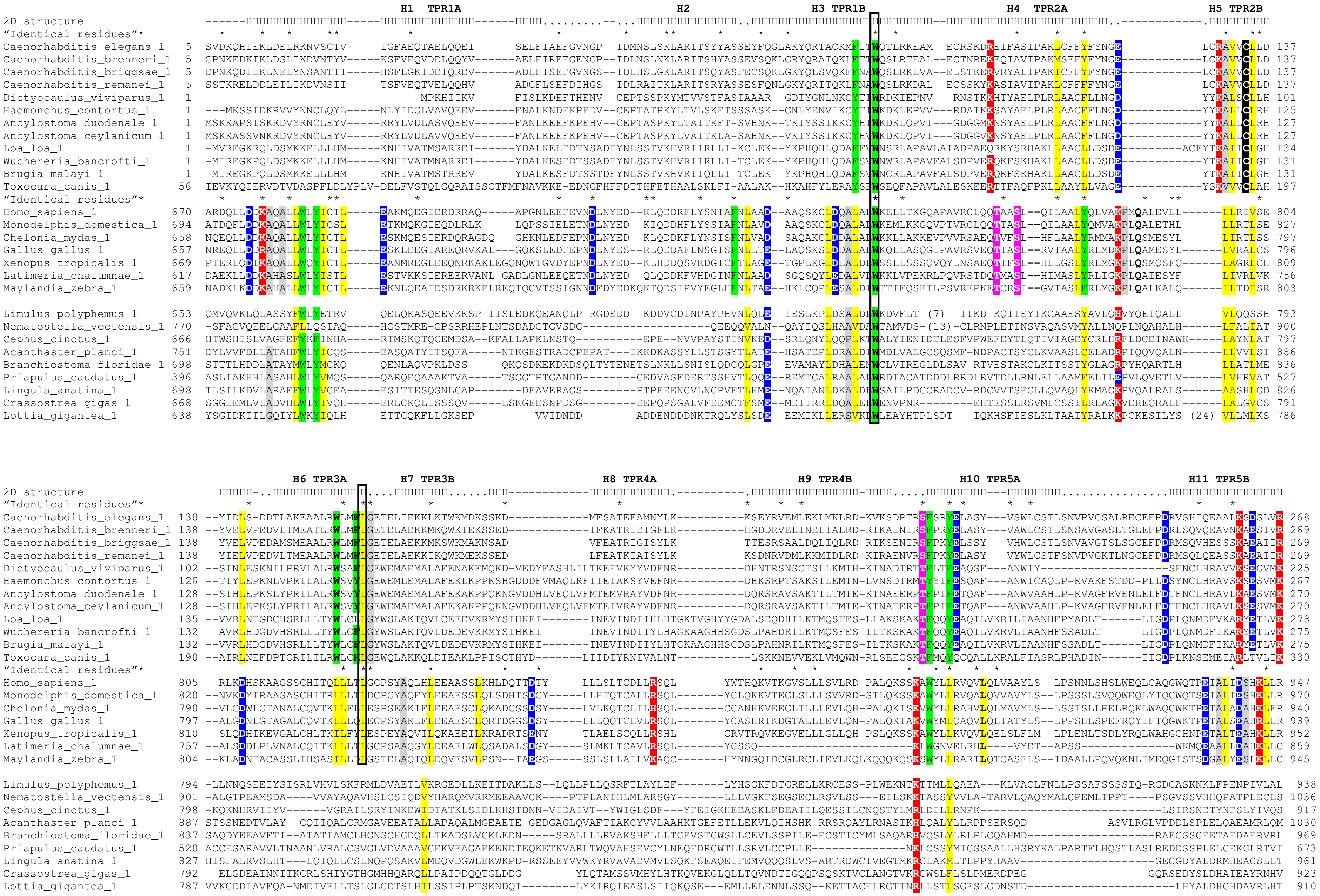
H1 TPR1A
H2
H3 TPR1B
H4 TPR2A
H5 TPR2B
2D structure
"Identical residues"*
Caenorhabditis_elegans_1 5 137
Caenorhabditis_brenneri_1 5 137
Caenorhabditis_briggsae_1 5 137
Caenorhabditis_remanei_1 5 137
Dictyocaulus_viviparus_1 1 101
Haemonchus_contortus_1 1 125
Ancylostoma_duodenale_1 1 127
Ancylostoma_ceylanicum_1 1 127
Loa_loa_1 1 134
Wuchereria_bancrofti_1 1 131
Brugia_malayi_1 1 131
Toxocara_canis_1 56 197
"Identical residues"*
Homo_sapiens_1 670 804
Monodelphis_domestica_1 694 827
Chelonia_mydas_1 658 797
Gallus_gallus_1 657 796
Xenopus_tropicalis_1 669 809
Latimeria_chalumnae_1 617 756
Maylandia_zebra_1 659 803
Limulus_polyphemus_1 653 793
Nematostella_vectensis_1 770 900
Cephus_cinctus_1 666 797
Acanthaster_planci_1 751 886
Branchiostoma_floridae_1 698 836
Priapulus_caudatus_1 396 527
Lingula_anatina_1 698 826
Crassostrea_gigas_1 668 791
Lottia_gigantea_1 638 786
H6 TPR3A
H7 TPR3B
H8 TPR4A
H9 TPR4B
H10 TPR5A
H11 TPR5B
2D structure
"Identical residues"*
Caenorhabditis_elegans_1 138 268
Caenorhabditis_brenneri_1 138 269
Caenorhabditis_briggsae_1 138 269
Caenorhabditis_remanei_1 138 269
Dictyocaulus_viviparus_1 102 225
Haemonchus_contortus_1 126 267
Ancylostoma_duodenale_1 128 270
Ancylostoma_ceylanicum_1 128 270
Loa_loa_1 135 278
Wuchereria_bancrofti_1 132 275
Brugia_malayi_1 132 275
Toxocara_canis_1 198 330
"Identical residues"*
Homo_sapiens_1 805 947
Monodelphis_domestica_1 828 970
Chelonia_mydas_1 798 940
Gallus_gallus_1 797 939
Xenopus_tropicalis_1 810 952
Latimeria_chalumnae_1 757 859
Maylandia_zebra_1 804 945
Limulus_polyphemus_1 794 938
Nematostella_vectensis_1 901 1036
Cephus_cinctus_1 798 917
Acanthaster_planci_1 887 1030
Branchiostoma_floridae_1 837 969
Priapulus_caudatus_1 528 673
Lingula_anatina_1 827 961
Crassostrea_gigas_1 792 923
Lottia_gigantea_1 787 910

```
                                                                                                 H12 TPR6A               H13 TPR6B                             H14 TPR7A               H15 TPR7B
2D structure                        HHHH.........---------..........-------........-------HHHHHHHHHHHHHHHHHHH..........HHHHHHHHHHHHHHHHHHH...HHH---------------HHHHHHHHHHHHHH.........HHHHHHHHHH
“Identical residues”*                                                           *             *                         *                       **     *      * *           **
Caenorhabditis_elegans_1        269 NRIPGLASSQFDN---------SVNASIWPFL-------DGHQED-S-------NYYVHIGSTIAWHFEMRRE-CALVNVTTAQTRDSMSAMILNLRVALKSASFFRV---------------LQTTNTLAYYSSIIEEAGSEKNAKLMRVSCVNLL 385
Caenorhabditis_brenneri_1       270 NRIPGLAAYQFDN---------SVNSSIWPFL-------EGSRNGSS-------TDYIHLGSTLAWYFEMRRE-LALVNVATAQTRDSMSSMILNLRIGLKSASFFRI---------------LQTTNLLAYYTALVEEVGSERNAKLMRVSCFNLL 387
Caenorhabditis_briggsae_1       270 NRVHGLAAYQFDN---------SVNASICQFL-------NEGSHG---------TDYVHLGSTVAWHFEMRRE-LALVNVAAAQTRDSISAMILNLRIALKSASFFRI---------------LQMTNVLAYYTGLVEEVGCEKNARLMRISCVNLL 401
Caenorhabditis_remanei_1        270 NRVPGLAAYQFDN---------SVSTSIWPFL-------EEKHHGSS-------TDYIHLGSTVAWHFEMRRE-WSLVNVSTAQTRDSMSSMILNWRIALKSASFFRI---------------LQMTNLLAYYTTIIEEVGTEKNAKLMRISVVNLL 387
Dictyocaulus_viviparus_1        226 NRIPGIHKESE-----------SIDFR--------------KVTCDT-------TDFVKACASYAEFCELRRE-YSIELLNVGMVHDAAFHSQLGLWSALKIGVFFRI---------------QQFVNVNTLIRQCVVQVEFRKDLQFCMNIIRILY 333
Haemonchus_contortus_1          268 NRLPGIQKEHL-----------PMDFK--------------CVARDP-------LDFMKACSSFAECCDLRRD-YVIELLHVGTIRDAAANSQLGLYCALKSGVLFRI---------------QQFVNTNTLLRSCVVHPDFKNDLHNCLDMLRMMY 376
Ancylostoma_duodenale_1         271 NRMQGIQKEDV-----------PLDLK--------------KIASDP-------LEFLKACASFAESCEVRRD-YAIELLSVGMIRDAAAVSQLGLWCAVKAGVLFRLIKQLAVDASHPTSTILQFVNINTLIRACVVQQEFKKDLRTCMDIMHTIY 394
Ancylostoma_ceylanicum_1        271 NRMPGMQKEDV-----------PLDLK--------------KIASDP-------LEFLKACTSFAESCELRRD-YAIELLSVGMIRDAAAVSQLGLWCAVKSGVLFRI---------------LQFVNINTLVRACVVQQEFKKDLRTCLDIMRTIY 379
Loa_loa_1                       280 CRSSVYYGSDMKEEDAGCAAFAGGEFQETKLMSTVSSKLQPQFINIF-------DEYLKLSAVLAEHFKVVLQ-LCFKYIELGILRESESEGLKLLHQVLKTTNLQRC---------------LAVLNVLFLTSTLAHGNSVNVRPDPFRPVIASLW 427
Wuchereria_bancrofti_1          276 CRSSVYYGNDMKEEDVGCAAFAGGEFQETKIDETKI------------------DEYLKLSAAVAEHFKVVLQ-LCFKYIELGILRESESEGLKLLHQVLKTSNLQRC---------------LAVLNVLFLTSTLARGDIMNVRPDPFRPMIASLW 393
Brugia_malayi_1                 276 SRSSVYYGSDMKEEDGGCATFAGGEFQEVNP-----------------------DDFLKLSAAVAEHFKIVLQ-LCFKYIELGILRESESEGLKLLHQVLKTSNLQRC---------------LAVLNVLFLTSTLARGDITNVRPDPFRPMIASLW 393
Toxocara_canis_1                331 NRYQAFFNYGE-----------GIELQ---------------KKVNP-------DDFLKLCSHISEHFEATIL-QCYELAETGVLRECEGIVMSLWRESFRLGSLPRA---------------LIAINLLMMLVMMS--NYMRKREQSLRQVYAALL 437
“Identical residues”*                                                         *                  *                               *        **               *              * *
Homo_sapiens_1                  948 SIILLLMGSDI--------------------LSTQKAAVETSFLDYGCSEKLVCENLVQKWQVLSEVLSCSEK-LVCHLGRLGSVSEAKAFCLEALKLTTKLQIPRQC---------------ALFLVLKGE------LELARNDIDLCQSDLQQVL 1055
Monodelphis_domestica_1         971 SIIILLMGRDL--------------------LFVQQSAVDAAFLDYGCSEKLVTENLVQKWQVLAEVLGCSEK-LVTLLGRLGTVSEAKAFCLEALRLAMKLQCVRQC---------------SLFLVLKGE------LELARNDLDLCQSDLEQVL 1077
Chelonia_mydas_1                941 SILLLLMGGDL--------------------LSSPKTVTETQFVDHGCSEKLIAENLLQKWQVLADMLVCSEK-LIALFSQVEMVCEAKAFCLEALKLAMKLQAIRWC---------------AGFLVLKAK------LELQRSELELCHSDLQQAL 1052
Gallus_gallus_1                 940 GIVLLLMGSNV--------------------LGSHKSAADIRFIDCGCSEHLVVDNVLQKWQVLADLLGCSEH-LVVLLSRVEVMCKAKAFCLEAIKLAVKLQAVRWC---------------ASFLVLKAQ------LELQQSELELSHWNLQQAL 1051
Xenopus_tropicalis_1            953 SIVLLLLGNDV--------------------LASPKGSSEVRFVDNGCTHDLVKENLLQKWQVLADLLSCCRK-LVHTLGRMGCVSEAKSFCLEGLRVAKTLQSMRHC---------------ADFLVRKAE------LETLRSETQLCEEDLQYVL 1064
Latimeria_chalumnae_1           860 SIVILLTGKSI--------------------VPSHKANANIRFMDQGCCRKLVHENLLQKWQVLADLLSCTHD-LVKLFGWVGSVCEAKAFCLEALKLTMKFKLLTQC---------------ADFLVSKAE------LELQRSRSDLCHLDLQQVL  971
Maylandia_zebra_1               946 SLLVTLVGKGL-----------------YGTTTSSNTNVVSSFINQGCSKKMVSDNLALKWQLLAELLNCSKK-MVSVRSDCGAINDARLQCLEALKLAIKLQTLSQC---------------SELLVMKAE------LELMQGEKDESRTDLDIVR 1060

Limulus_polyphemus_1            939 DAWKLCMGLI-----------------------KYLSSAEAQKSYKD----YLSYKPALLHTLLSSTIL-VGQ----LYIDIGAPREARCYLKDTLKVAQEM-----------------------ALAYRSAEFLLTLANLDAVCENINDCKVKLNG 1040
Nematostella_vectensis_1       1037 DGCTWVEVAATAIFGQSLL---------------DVDSTAVCKVGSD-----ECLRLTTLHILLHCQLQ-LGA----MLTRLGVVKQAAKFLADGLALAGRF-----------------------HLPYRVIEFKIFQSELDIQIHKLDRARSVLLG 1145
Cephus_cinctus_1                918 LYAMISLS----------------------------SNLIETKSESK-------ERILYGYDVLLSSTNRLSL----RLNSLLSFKEISAHLIQRLKISQKL-----------------------GATLRVAECLKNLCFIDLSRMQLDDCEVKLQG 1012
Acanthaster_planci_1           1031 GVGRLMFGEGVFGNEQ-------------------SLQGEGFKLAAVGNDSGGNDLEFNQWTVLANVLRSLYD-VGSLYLEQGCTREAKSYLKEGLLLA------------------------DKFMLPRRSVAFLLQLSNLHVQCGQEDDCQASLS 1143
Branchiostoma_floridae_1        970 SLARLLFGDRFKETDRLVT---------------ASSASNSAKTKRGY--------PHLPWWCCDDFLTSLLD-MGKLYSDQGCVKEAKSYFLEGLEIA------------------------ANFKLPRRCAQFLTMLAEVELHRGLTEDCQLLLK 1078
Priapulus_caudatus_1            674 GIAKLMLGRD------------------------AAEAAAGDDDVACV---DRTTRLVTRYRILIDMLCALHQ-AGDFYLHCGLAREAKTYLKEGLTYA------------------------ERFGLTKWCARLRLSLCEIELRSGQPLDCELMLD  778
Lingula_anatina_1               962 GVWKFLQGK-------------------------------ASESAS----------LMDKWSIMTEYLDTLQA-TGHMYLDIGESRAAKCYLKEGMILA------------------------QEFNMPRRLSGFLLSLAKLDTLSEKFTDAEAKLN 1052
Crassostrea_gigas_1             924 SVVYFLLGKEHWAV--------------------------DNKGLN----------TTEKYDIIQEYLLSLYH-LGQLERLVGDTRSAKFHIKEGWTTA------------------------HFMGLPKWASMFMNEFSIIHILSENSTAAISSLT 1019
Lottia_gigantea_1               911 SVNHFFLEK-------------------------------KSKAGS----------PHQKWSIIIEVLESLLN-LVKLYRYIGDDSMAKCYAREGIKTA------------------------HSLLLPRWCLLFKFELCRIYSITCCLKEAQDVLT 1001

                                                                              INSERT 1                                                                                   H16     H17 TPR8A
2D structure                        .---------------------------------....----.................-.......---........................-----------------------------...........HHHHHHH..HHHHHHHHHHHHHH
“Identical residues”*                                                                                                                                                      * *  *    *            *
Caenorhabditis_elegans_1        386 S---------------------------------SNPI----IVRCSTPKETGATSRAHTPMAGSSV---SEKQNTMRPDLADLLGDLELLDEQ-----------------------------SFHPITRSCVCNVCTIYPLHSSFAAEYMMSYAIH 473
Caenorhabditis_brenneri_1       388 S---------------------------------SEPV----IVRCSTPKEPRVPSRAHTPLPGEKI--RPQQQSTMIADESDEFEDIYLL-EK-----------------------------PFHSITRSCSCTVCLHYPNSSTFAAEYMLSHCIY 475
Caenorhabditis_briggsae_1       402 S---------------------------------SQPI----VVRCSTPKETRAASRAHTPLPGEESGNRRSKSDT-LNILADELEELDLYNDQ-----------------------------IFHPITRSCNCNVCLHYPLSTTFAAEYMMAYCIN 491
Caenorhabditis_remanei_1        388 S---------------------------------ANPV----LVRCSTPKESRGPSRAQTPLPGEKSGYVPHISETMVAPLEDLMEDLDLFDEQ-----------------------------PFHSVTRTCPCNVCLHYPLSCTFAAEYMMSYCIH 478
Dictyocaulus_viviparus_1        334 LKEPSSKN-----------VFGGSKQK--EVLPDDSEM--SLLLTAKSPMGNRLFPASE-------------GQKSVAISIMESFEDLRLE------------------------------------------------------------------ 397
Haemonchus_contortus_1          377 SNGSSMTDM----------LFGCSKKRPPE-VSFSDELTTEIPLPRISTP--SRSPARV-------------GQKSLATTLIESFEELRLETGKTPQRGRSPSRIASKVIDSFHELTLSETVSSLHELSDSCSCNFCWFCRTNYNASFEYWFSSFLY 507
Ancylostoma_duodenale_1         395 SSDTLLSKT----------SLGCTKKKPPEMLEFVDNI--DLPIPCTSTPIRSKSPGRV-------------SHKSVALSLAESFEELRLESAKTPPRCKSPGRITSKFIDSFHELTLSETICSLHEISEECECNTCELCSSNYNAAFEYWMSSFLY 526
Ancylostoma_ceylanicum_1        380 SSENLSSKT----------SLGFTKKKPPEMLEFVDNI--DLPVPCTSTPIRSKSPARV-------------SHKSVALSLAESFEELRLESTKTPPRCKSPGRITSKFIDSFHELTLSETICSLHEISEDCECNTCELCSSNYNAAFEYWMSSFLY 511
Loa_loa_1                       428 NRSRNET------------SVCVDKEM---------------------------------------------QNKKLNLSSKDQKIIDLTEDEPNNLPQANKENMENKL--------------ILEEHVEYCDCVICTLCDINPQMHIEAIFSKMLF 513
Wuchereria_bancrofti_1          394 NRSKSGI------------SVCTDSER---------------------------------------------LNRELNLS--SQKIIDLSEDEPNNLSQANKENMESKL--------------ILEEHVEYCDCVICTLCGMNPQMHIEAIFSKMLF 477
Brugia_malayi_1                 394 NRSKNGI------------YVRTDNER---------------------------------------------LNKKLNLSSGSQEIIDLSEDEPSGLPEANKKNMESKL--------------ILEEHVEYCDCVICTLCGMNPQMHIEAIFSKMLF 479
Toxocara_canis_1                438 NVPKRSD------------DSSNTAER--------------------------------SPDPGTLA-------------KEPSLAALMLHAFCYGQESGDGEKKEEKIEDD-----------KLESHNEKCICVVCATCEHDLQLQFDISFAAFLF 526
“Identical residues”*                   **                                                                                                                                 * *  *    *            *
Homo_sapiens_1                 1060 FLLESCT------------EFGGVTQHLDS-VK--------------KVHLQKGKQQAQVPCPPQLP----EEELFLRGPALELVATVAKEPGPIAPSTNSSPVLKTKPQPIP----------NFLSHSPTCDCSLCASPVLT-AVCLRWVLVTAGV 1170
Monodelphis_domestica_1        1082 FLLGSST------------EFEGIAQLPNT-VK--------------RIHPQKGQQEPRIQPGSELS----EEEAFLKGPALQLVATVEKDPGP----SVSSPVLKSKPRPCP----------AFLSHSLSCSCILCTDPALV-VVCLRWLLVSAGA 1189
Chelonia_mydas_1               1053 FLLESGTGEVSFTSDLKERNFETKEKQKSE-VKI-------------KPKKGRSKGSKHQGPSAEPA---GEEDGFLKGPSLEFVDTVSRQEK--ESVLTTSPVLKPKKKRCL----------NFLSHSALCSCSLCSDVTLS-AVCLHWVVSYGQW 1175
Gallus_gallus_1                1052 FLLESGT------------EFEADEKQRAP-LKI-------------LPRKGKPEGRKQRDEGSELP---GEDEGFLKGPALEFVAMVSGLEK--ADDLSSSPQLKPKRKRRL----------AFLAHPAACPCCLCSDLTLS-SLCLRWLLCCARG 1162
Xenopus_tropicalis_1           1065 FLMESCT------------DFAAKSKQK-E-VKI-------------KLCKGKPFHKVDITETPPSP----PKDDFLKAVDLHYVETRDLSKAP------ESPTCKPSLEKNL---------PHFLSHTQDCICPLCSDLVLS-LLCTQWLVAKAEN 1170
Latimeria_chalumnae_1           972 FLIESCT------------DFDSRGQVQGK-MKI-------------KVKKQKAVRKQTTKTDPTAE----GEDAFIKGPSLRNIATVSRDK---EGALTASPVLKSKVRKQL----------SFLSHQPACWCLVCTDVELS-LACTKWTVVFGEA 1080
Maylandia_zebra_1              1061 NLLERST------------DFSDQVQRA-E-VKI-------------KPRKGRPVQRPQSPLP--------TIEDDLK----DILSTRWTSKEPIVKDLSGSPPLKALPHRWL----------SSLTHEPECQCPQCSEPCLG-RATARWAAAQADL 1163

Limulus_polyphemus_1           1041 -VEYILHTSLP----------ATSVNS---------------------EAAREKNELSEGSDE-EDFLVRRPGRSLRGTVREQLLHG-----------------RRETESSPISQQHVKTSIPTQWEHSISCDCNLCQHIKLHSLELEIVGLQAYCL 1147
Nematostella_vectensis_1       1146 -VASQMAPLFH---------------------------------------------------------------------------------------------------GTTDRLEDLTDVHAVVSHKDTCRCMCCSDPALQAIQIRVLLVLARFF 1202
Cephus_cinctus_1               1013 -LEHILDIESI----------ELSMKS----------------------------------KLQTVETSSYSVSSSERMVESVRD--------------------IAQNPASPVLRRKVFAPSDFLLH-KDCICYKCRNVPYQYLVFASTYIRAQLY 1103
Acanthaster_planci_1           1144 KVQQILHL--PEKAAQARWRGASMVTQDERKSSS----------------GSETHEETDDDNFIRTKRISISAECSSTTWRLEDTDTSS----------------------SPWLKAQTVKLPSYHSHPSDCLCASCADVSLHAQDMTSFLRLAQRL 1260
Branchiostoma_floridae_1       1079 SACSVLAS-(46)-EKERKRTQNKKDARRQRKSAGCFGFD----------VLESEGSEDEDDNFIRSKLI------SVSQQLEEDDGIDSDLEDYGK-------------HQPSFPTM--QLPDYVSHNANCICSVCKDVVSQTSYLDVFLTATECA 1244
Priapulus_caudatus_1            779 SVDYTLNA-EPISAPPLVATVTVATATPANPTTAGRKKKAASGKAAGDTGKMLRGCRGGKPSSGATATPF--------TIYTDDASAADDFLISSRPLSSPQGAP-PSNAAASSPALSRQTRRGGAEHAAGCACARCGDVALQLLNALASALRARCL  925
Lingula_anatina_1              1053 MVHFILNSEVH-TVKPEKQPTRKMDVENEKAEKQQKKKDDCFGFDISDEEDDDSEKGLDFLKRKVL----------------EDPIPSDENDSGNEDPCA-----------SPSLRKHESKLPVYLSHSNKCSCVLCHDFILHPLSMEYIHGLACLS 1181
Crassostrea_gigas_1            1020 KTCQVFNGSQDTAGENATNRSSKSRKSKTAGSFGFD----------------------------------------------DDVEKEEDSVESSQSPYP----------------------PCWQHLTDNSICDVCQDNTVKLAVLSYALVTAKFE 1108
Lottia_gigantea_1              1002 DIASILYSNKAEKPGLTKLVSYNTNTKNIPLKDMAEYLQRKRSFADDCFGFESIRDAGIDRFSV--------------SDVSTDPLCDDIPDIN------------------------------LPDHDSSCKCIACKDNSLLDLTSKYWFVLAETC 1114
```

```
                                     H18 TPR8B                                               H19 TPR9A                  H20 TPR9B                H21 TPR10A
2D structure                  --------....HHHHHHHHHHHHHHHHHHHHHHHHH---------.....-------.....HHHHHHHHHHHHHHHHHHHHH.....-----HHHHHHHHHHHHHHH....------..HHHHHHHHHHHHHHH...-----------..
"Identical residues"*                    *  *   *        *        *                     *    **                 *      *           ** *               * * * **
Caenorhabditis_elegans_1   474 --------SDFSQLSIKHFNDEFARIRERGMSSQVLM---------HRDSSV-------RPRPNIIQNEIFGMCVIRWLTKKLDSKESAD-----EDTMEIFNNALKIVRYLQQ------RTTDMILAVTQLGRQLEFPM----------EC 580
Caenorhabditis_brenneri_1  476 --------MNFSQDAIKHFNDEFSHIRERGMEFQLMM---------HRDRSV-------RP--------------------------------------------VVKYLNL------RTTDLLLAVTQLGRQLEFPF----------DV 541
Caenorhabditis_briggsae_1  492 --------SDFTQISMKHFNDEFAQIRERGMNAQTMM---------HRDKSV-------RPRPNIIQNEVFGLCVIQWLKRKVDTKQLVD-----TETLEIFKNALKIVKYLNL------RTTDLLLAVQQLGRQLEFPQ----------DC 598
Caenorhabditis_remanei_1   479 --------SNFNQTSLKHFNEEFIRIRERGMSSQTMM---------QRNTQV-------RPRPNIIQNEVFGMCVCQWLVRKIDSKQYVD-----QESLEIFNNALKIVRYLNI------RTTDLMLAVQQLGRQLEFPQ----------DC 585
Dictyocaulus_viviparus_2     1 ------------------------------------------------------------------MCETFAICVVRWLRRLEEACWNRENAALRLLREKIVQDALKICSYSSL------RTVPYRLAISQLERLQNLPQ----------RL  70
Haemonchus_contortus_1     508 --------ESMSESAIQCLEERFHALRARVAPEQCAV----------LGKKV-------KPRPPSIMCETFALCVVRWLRRLEDSSWDSENLKLKPLRDSIVKSALKICGYSSV------RTVCYRLSISQLERIRKLSR----------CY 618
Ancylostoma_duodenale_1    527 --------ESMSNESIQCLEKQFQILRARVAVEQRAV----------LGKKV-------KPRPPSIMCETFAMCALRWLRSLEDSEWDHDNSSLKALREQIIASTLK-------------------------------------------- 608
Ancylostoma_ceylanicum_1   512 --------ESMSNDSIQCLEKQFQMLRARVAVEQRAV----------LGKRV-------KPRPPSIMCETFAMCAVRWLRSLEDAGWDHDNSALRALREQIVASTLKICGYSSV------RTIYYRLAVSQLERIRRVLR----------PS 622
Loa_loa_1                  514 --------YRYSSRMFRVLSERILKLPQETALDVIAICSSLQKQ--ATDKAIE------ERRYPLAALDLFLLAAIRWLRRVGEEEK-------PEIIQDVIRESLKLCSTDPI------YFRWQWLTIRQLARTPL--K----------IP 624
Wuchereria_bancrofti_1     478 --------YRYSSRIFRMFSERILKLSQETSLDVIAICSALQKE--ATDKAIE------ERKYPLEALDRFLLAAIRWLRRVSEEEK-------PEIIQDVIKESLKICLTDPI------HFRWQWLTIRQLARTPL--K----------IP 588
Brugia_malayi_1            480 --------YRYSSRMFRMFSERILKLSQETSLDVIDICSALQKE--ATDKAIE------ERKYPLEALDRFLHAAIRWLRRVSEEEK-------PEIIQDVIKESLKICLTDPI------HFRWQWLTIRQLARTPL--K----------IP 590
Toxocara_canis_1           527 --------DSFSSHSFQTLATKFRTLETKFARAHSCM----------------------------------------------------CNAIGACVDAELCELEPC------HHRWQWLTIRQLEREPMIDR----------TS 595
"Identical residues"*                      *  *   *       *       *                      *    **                  *      *                 ** *                     * * * **
Homo_sapiens_1            1171 --RLAMGHQAQGLDLLQVVLKGCPEAAERLTQALQAS---LNHK--TPPSLV-------PSLLDEILAQAYTLLALEGL-NQPSNES----------LQKVLQSGLKFVAAR---IPHLEPWRASLLLIWALTKLGGLS----CCTTQLFAS 1290
Monodelphis_domestica_1   1190 --ELSSGHQAKGLDMLRSVLRRCPGATHHLAQVLQAT---LSQE--ASPSPA-------PGFLEDLIAQAYAHLALEGI-NHPPDKG----------LWDILETGLNFVASR---TPFLNPWHASLLVTKSLATLSSLS----CCTTQLFGN 1309
Chelonia_mydas_1          1176 --ELASGNRAEGLGLLQASLERCMVMTSRFSTMVREA---SQQK--GKKAAIWDRSM--LGLLDNLVANIYATVAAQSMGGHQPEKR----------LWELLEAGLTFLSSRVPCLPGLEYHKASLLLTKAVATIYSLASKHNGCMAHIFSS 1308
Gallus_gallus_1           1163 --ELAAGSAAEGLGLIQATLQRCGTVGPRFTVLLQDK---LGSG--HKAPA--------LRLLDDLVATGYAALALHSLAGLCPEEK--------- LEEHLKAGLSFLASCSLHLPSLGVSKASLLLAKATAAVCRLAAKHGGSVDAVFAS 1289
Xenopus_tropicalis_1      1171 --------NTQSRGLLKSALKRCKALTQRFSGIVQEI---LGKG--VTKPAV-------LGVASELMARVYLAMA-----SQSPSKL----------PEETLEEGLSFVSSR--QATLPKHWRAGLLLAKALCSIYNLAAKHGGCVADMFVQ 1285
Latimeria_chalumnae_1     1081 --EAAAGNHAECRRLFQTSLERCETLAERFAGFIQGT---LAKEKPAKSASLKGKGMPSFGFLDDLCAKIYSKMAAFSL-TPKPEKT----------TWKVLNAGLIFLTSKAVCLPSLEHAKAALLLAKAVALISSLAAKHCCSATELFSP 1216
Maylandia_zebra_1         1164 LLQLDPNDTSVSSKLQWATLSRSKTVTAKLGVKLAKL---FPLY--NTGKHI-----SKPSLMQDIVGRVYLHMALSGV-DPSLTKVCA--------IWKILEAGLAFVESTP--SPMLRPVRAGLMTAKAIASLISLAAKNECTPEELFSK 1284

Limulus_polyphemus_1      1148 AVMGSRSRAIRWLK---NILKLVTFLADKVDF-----------------------------LLTSAVKMI-------------------------------------------------------------------------------- 1185
Nematostella_vectensis_1  1203 QASAAPKVSVNCLE---GAEVLCNNAVARSKR-----------------------------TLDEITNML---RLGVSEPC---ETDKAPRTKSKGKSKKAKKDDSAECPITTA-----VTAKAMYGVYFA-----EVFVLNAEILLSRGKLT 1307
Cephus_cinctus_1          1104 ALQHMTSAAMEHFY---GAFKIRQSIFARESQGSLDSTINKM-----DLERHPWEFYYN-MVEYVLFLL---DFSRF------------------------------------IKEYKLAK----------------------EE 1178
Acanthaster_planci_1      1261 ELQGEPTQALANLRVALDI-GDWSATKTPSLL-------LRSSSCLHND------EGVKQTKGPEMKLARPSRSSGGQVNLHARATTEIHVCIT----RLTLLNGNLSEAEHTIAGGLELSIDENI--------PKAWLHFYRILVSLELKA 1386
Branchiostoma_floridae_1  1245 LSEGLPAVAIATAAASQKIHGKVCMCVSSTMK-------DFSSSLIHGKKKET-----------------KTKKNSNTEHLFAPQLALLHCRLS----EAYLMQENFSKANTNIESGLLLFQGKSRAIH-LHSNTQAHLLYNAALCAILQEA 1367
Priapulus_caudatus_1       926 HDQSRDGELLA---------GRFAAARARA------------AAHAHVE------GATATLRDVVVERLGVAPGGAPSGSVLAESGAAYHVVVA----TAQLRGDDIGSAEAELARGQQLLAGHHG-------ELRARLLHLEAVLSIRKEA 1039
Lingula_anatina_1         1182 MEMFAVSECATLVKTGLQF-YNTAFQKSTQT--------LMSKSCDNLY------KGKKEIDKLVESL-------------FLPWSVRFYIQVADTKIETNLQWGPAGRA---LARAASHLNCSTATGLVNLWKERAFVFYLQALVGLKGMA 1302
Crassostrea_gigas_1       1109 ---IYIQQNLTFEK--LD---RINCICERFILQ------EPSTSKLGRDLPFCCFQDNSELKDVYVEC--------------------LSLIA----ETSLLLGKTEKCAIVIETAQKFFQSKYVSDLVANLIQNKFTVFKAQMCLMRSLS 1221
Lottia_gigantea_1         1115 FHNKFYQHAETFLRKILQ---RSSEIRPKLFENLLTIEKRLCESNQMMD------KVPNVFNDILYES--------------------YCLLS----ECSVYLNSTHETQEICQKALDILDSDVRCSV-----EQNLLRARVNMCLVQLQM 1227


                                         INSERT 2                H22 TPR10B           H23 TPR11A                  H24 TPR11B      H25
2D structure                  .................--------------...HHHHHHHHHHHHHHH.......HHHHHHHHHHHHH.----------...HHHHHHHHHHH...HHHHHHHHHHH...
"Identical residues"*              *   * *                                    *                   *                               *            *
Caenorhabditis_elegans_1   581 NYSWMRPTIRKPRVKAT-(29)---------FDKERFEKVRLAMRNEMNHYGHILYREWRCRLFAYVGRT---------SRDPWEAAYAWAESTQIGARNAVQSRLEKCKR 697
Caenorhabditis_brenneri_1  542 NYSWMQPIVKKPRSKKG-(30)---------VDKEKLLKIRSEARREMNNYAHILYREWRCRLFPYIGRI---------SNDPWEAAYAWAESTLIGSRNAIQCKLERCRK 657
Caenorhabditis_briggsae_1  599 NCSWMHPIVKKPRVKAR-(30)---------FNKERYDKVCHEIRREMTQYTHILYREWRSRLFPYVGRT---------SKDPWLSAYAWAESTLIGSRNAVQCRIAKCRK 729
Caenorhabditis_remanei_1   586 DYSWMQPVIKKPRVKAG-(30)---------FDSARYEKIRTEMKLEMNLYGHILYREWRCRLFPYIGRT---------STDPWEAGYAWAESTLIGSRNAIQCRLEKCRK 701
Dictyocaulus_viviparus_2    71 RYDWMEDDTRTNNFMNT-(27)------NSAEQKDLTKGIVDDALKEYYRYSGLLYRDWRFSLCTFLGEY---------ATCPWLAAMMWSESTLLATRQLARHISGKFKG 184
Haemonchus_contortus_1     619 RYEWMKDTSRAGKFMDA-(23)------EVVAQKIVEKNMMKEALSEYASFSNLLYRDWRFPVCTFLGEY---------VSCPWQAAMMWSESTMLATRQLSRFMSGKMNG 730
Ancylostoma_duodenale_1    609 ------------------------------------EEQKKIEEAAIAEANAEYASYSNLLCRSMGSSNDVVRVN------------------------------------------------ 647
Ancylostoma_ceylanicum_1   623 SHDWMTDPSLTNRVIST-(21)------DSEEQKKAEEAAIAEANAEYASYNNLLCRDWRYPMCTFLGEY---------SKCPWEAAMMWSESTLLTTRQHSRLLLGKMSG 732
Loa_loa_1                  625 KLEWMHLHVSPI--KAG-(41)--NHKTVTNVTPSYIDMEMEEARKDFNSFSHLFYREWRFQTCSYLGQVCAFQYKADWIEPGWSAAYYFNEAMFISTRQLARMIYKKSEE 765
Wuchereria_bancrofti_1     589 KLEWLRLHVKARLRSPF------------------------------------------------------------------------------------------------ 605
Brugia_malayi_1            591 KLEWLRLHVSPV--KAG-(33)--NRNTVTNVINNYVDTEMEEARKDFDSFSHLFYREWRFQICSYLGQVCAFQYKANWIESGWSAAYYFNEAMFISTRQLARMISKKSEE 733
Toxocara_canis_1           596 HLPWMLPSVSSDRIRLS-(45)--DSETA-NGMNPRYCQLYETANEDFEQYSHLFYREWRYLNCSSLACA--------NLSDNWMSAYFFSEATCFATRQLARTVDEEGES 733
"Identical residues"*               *   * *                                       *                   *                          *            *
Homo_sapiens_1            1291 SWGWQPPLIKSVPGSEP-(262)-PSAPV-ATGLSTLDSICDSLSVAFRGISHCPPSGLYAHLCRFLALCLG-------HRDPYATAFLVTESVSITQRHQLLTHLHRQLS 1641
Monodelphis_domestica_1   1310 TWAWQPPAAKLSQMSKS-(256)-ASTPA-AFGQPTLESIYDSLSIAFRCVSHCPPSELYAQLCRLLALCLG-------HRDPHTTALLVTESMSITSRHQLLTTVHRHLH 1660
Chelonia_mydas_1          1309 AWAWKPLTPS-SETKGS-(322)-SSLAA-AVSLSSLDSVCNSLKAAFQAISHCPPGTLYCQLCRLLALCIG-------SRDPLTTAYLVSESVSITVRHQLMSNVHRRLY 1724
Gallus_gallus_1           1290 AWAWQPAPLSPTEPKAA-(361)-NVLPA-VDDASLLDTACGLLKSAFSCISHCPPGTLYSQLCQLLALATG-------DQDPITTAYLLAESVSVTTRHQLLSIIHKKLH 1745
Xenopus_tropicalis_1      1286 LWGWQPHQNGKKAADLN-(272)-SSLPA-TFSASDLSLVSSLLHEALESIAHFPPSTLYSRLCRLLALCRG-------RQDPYVTALLVSESVSITLRHQLLSDIHRKLR 1652
Latimeria_chalumnae_1     1217 LWAWKPFPIPDPAKEKA-(359)-SALTSDVADFSALDSVYACLCSAFHSISHCPPSILFSHLCQLLALCRG-------SCDPYTTAYFVSESVAITARHQMLTNIHKKLS 1671
Maylandia_zebra_1         1285 AWTWNMPKEGKDLKSEQ-(303)-SHADS-RPDNLTLEDVQSLLRSAWLTLQHFPPPTLYPTLCALLALTTG-------QQNSITTAMLHSHSVGITSRHRTIRHIASCLK 1692

Limulus_polyphemus_1           ------------------------------------------------------------------------------------------------------------------
Nematostella_vectensis_1  1308 KAR-DVTAAGLDTVEEV-(172)-DEDK--GDH---LQVAMEYLTRALDLTQPLPDPRLYSSICRVLALCYG-------RVSPSDAAHYSNQSLGVTLRQEALNSSRKKLR 1567
Cephus_cinctus_1          1179 KGL-NIALEAINICQTH-(143)-NINSERLSLEDV-----IHKASSLLPEMSSRLRKLSTGQSLRETPD--------ATKIQELIKVLEDSTIKDTARPIRKSRSRVKLE 1410
Acanthaster_planci_1      1387 RATETTVGDLTFSTWDL-(436)-IAQTGLSNLDTSLDSVIAGLEQISAQVCHRAPSLLYRRVCQLLAACHG-------DREPMKAAYYLSEAAAVTLRHQKLASLAKKMK 1919
Branchiostoma_floridae_1  1368 AKCNCSPFDVLNCNHST-(363)-SSSDDSEEKRQG------SKDISSKRQEAFRPTQAFHRRCRGAERVCADWRG-GGELAWVHTSYYQLHAGGFT----CTGGKDHTLW 1822
Priapulus_caudatus_1      1040 EGRACDPYSLFRVGYCE-(469)-LSAGVVRHREFSVASVKLLLDEATSLLRQHPPSPLYGEVMLLRALCDV-------TLDPRAAARHAAEAAAVTLRHQACLQITKKLR 1604
Lingula_anatina_1         1303 EPSDYNNIVSNLNDEFQ-(480)-EGEDDFEANFTDPEECIDLLNKAYNLIRQFPPMLLYRKVCHMLGLLLV-------ERDPTAAAFYMSEAVAVTFRHSLLASCNKKIK 1878
Crassostrea_gigas_1       1222 SKRFTEEEMDDLDEVTS-(509)-LNKDSLRADSPKEEDLTSMLEVAFEQARHLPTTGLYSSICNLLALQYL-------ELCPLKAAYYLTEGMVVTFRHQSLVNCSRKIR 1823
Lottia_gigantea_1         1228 SDLNLL-SLQEPAQFGK-(409)-VKRNQQSVTNPDNEEIIKKLEMSYQQVCHFPSNPYYPVICKLLALQYR-------TLSPYKTAFYLTESAAVTFRHQTLIDIGKKIR 1729
```
