# Supplementary figures and images for: Conservation of the separase regulatory domain
Source: Biol Direct. 2018 Apr 27;13:7. doi: 10.1186/s13062-018-0210-0 (PMC5921967; doi:10.1186/s13062-018-0210-0)

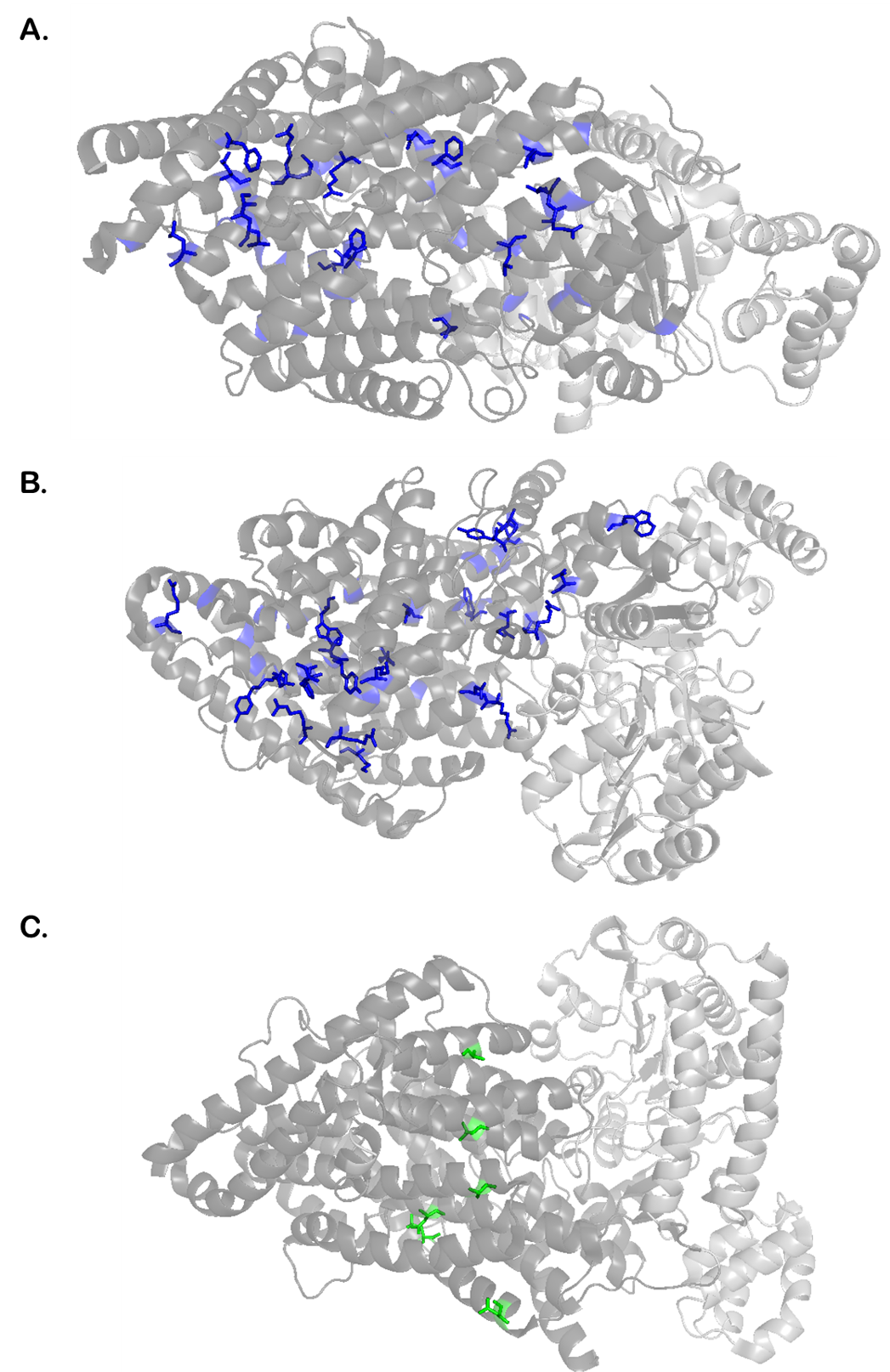

Supplement: Supplementary file 4 — Figure: Separase N-terminal residues conserved among nematodes are distributed throughout the structure. C. elegans separase Cryo-EM structure (PDB 5MZ6) illustrating N-terminal residues conserved among nematodes found in the interior (A) and on the surface (B) of the TPR-like N-terminal domain. Intragenic suppressors of SEP-1(e2406) are shown (C) and are not among the conserved residues. The structures are oriented with the N-terminus to the left with a perspective that best illustrates the distribution of each highlighted residue. (TIF 926 kb) [file 13062_2018_210_MOESM4_ESM.tif]

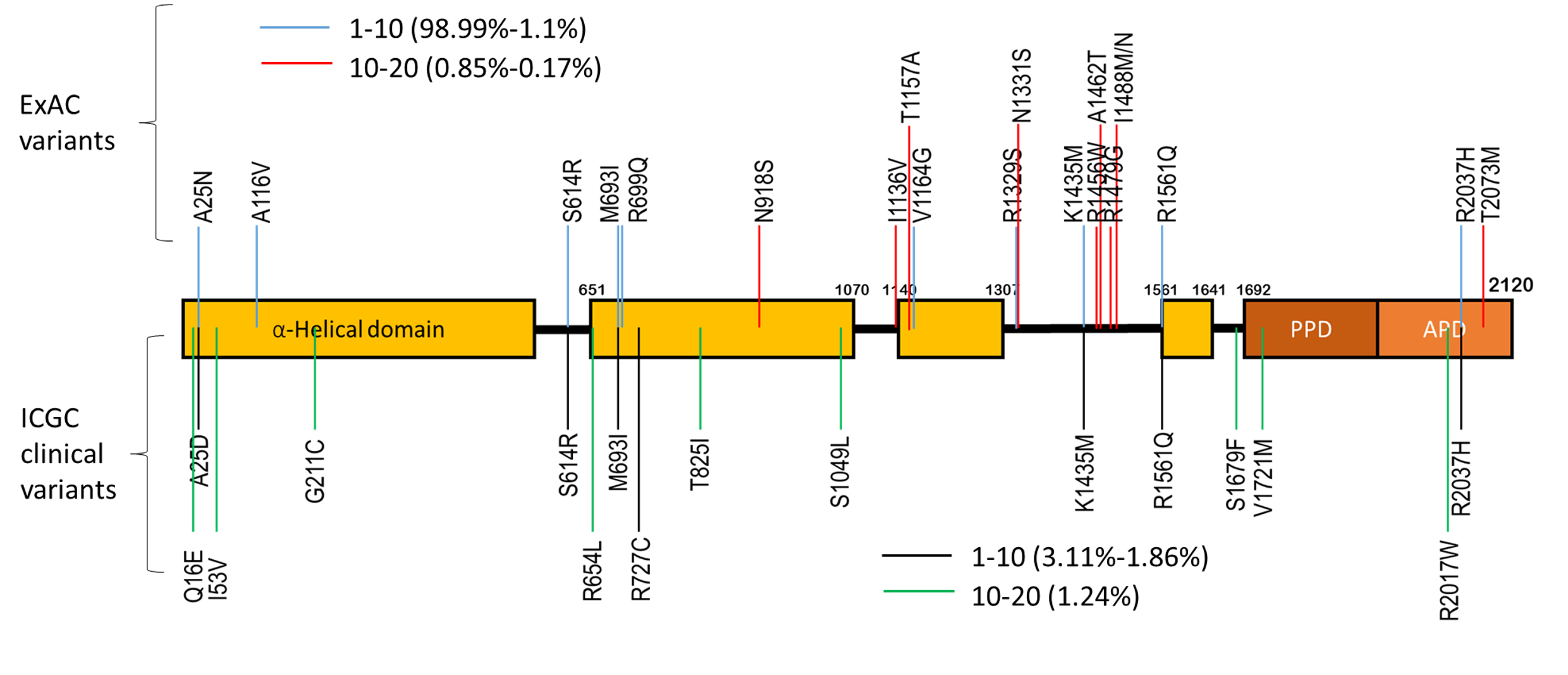

Supplement: Supplementary file 6 — Figure: Known mutations in human separase (ESPL-1). The collection of separase allelic variants of human Separase from the ExAC exome collection (http://exac.broadinstitute.org) and the ICGC (https://icgc.org/) which collects genomic sequences of various cancers. The frequency of each missense mutation is indicated. (TIF 413 kb) [file 13062_2018_210_MOESM6_ESM.tif]
