# Supplementary material for: Conservation of the separase regulatory domain
Source: Biol Direct. 2018 Apr 27;13:7. doi: 10.1186/s13062-018-0210-0 (PMC5921967; doi:10.1186/s13062-018-0210-0)
Supplement: Supplementary file 5 — Table: Conserved residues found within the N-terminal domain helices. Residues that are within interacting distance (as assayed by measuring a distance less than 6 Å (Å) between β-Carbons) are indicated. These residues are generally located within the same helix and don’t appear to be important for stabilizing inter-helix interactions. (PDF 29 kb) [file 13062_2018_210_MOESM5_ESM.pdf]

**Conserved residue**	**<6 Å β-C distance**	**Same helix?**
F90	W93 (5.5 Å)	same
W93	F90 (5.5 Å)	same
E127	R130 (4.7 Å)	same
L158	I514 (5.3 Å)	no
F220	Y222 (6.0 Å)	same
D264	None	
D330	R329 (5.1 Å)	same
F520	None	
W584	Y582 (5.7 Å)	same
R685	None	
